# Amino-Modified Mesoporous Bioactive Glass Adsorbed with Osteopontin Enhances Osteogenic Differentiation and Matrix Mineralization via the Erk1/2 Signaling Pathway

**DOI:** 10.3390/jfb17030153

**Published:** 2026-03-19

**Authors:** Ying Yang, Kunlu Lin, Zheng Zhou, Libangxi Liu, Long Liu, Haoming Liu, Hanyue Mao, Xiaoyan Wang

**Affiliations:** College of Science, National University of Defense Technology, Changsha 410073, China; yangying5952@163.com (Y.Y.); kunlulin@163.com (K.L.); zhengzhou23a@163.com (Z.Z.); cdllbx@163.com (L.L.); llbio@126.com (L.L.); lhm@nudt.edu.cn (H.L.); maohanyue22@163.com (H.M.)

**Keywords:** mesoporous bioactive glass, amino functionalization, osteopontin, osteogenic differentiation, osteogenic mineralization

## Abstract

Mesoporous bioactive glass (MBG) has been extensively studied in bone regeneration due to its excellent bioactivity and osteoconductive properties. Here, we prepared amino-modified MBG (MBG-NH_2_) adsorbed osteopontin (OPN) to form MBG-NH_2_/OPN composites, enabling the sustained release of OPN and enhancing osteoblast differentiation and mineralization capacity. Interestingly, we observed that MBG-NH_2_ promotes the formation of osteoid deposits and calcium deposition in vitro. Furthermore, we also found that MBG-NH_2_/OPN significantly enhances cell adhesion, differentiation, and mineralization. Consistent with these observations, we found the expression of the osteoblast-specific marker gene increased, including *bone morphogenetic protein 2* (*Bmp2*) and *Collagen I*. Intriguingly, we also found that MBG-NH_2_/OPN promotes osteoblast differentiation and mineralization through activating the extracellular regulated protein kinases1/2 (Erk1/2) signaling pathway. We concluded that MBG-NH_2_/OPN enhances osteoblast differentiation and mineralization through the Erk1/2 pathway. These findings indicate that MBG-NH_2_/OPN is a new potential biomaterial for bone regeneration.

## 1. Introduction

In recent years, bone defects caused by trauma, infection, tumor resection, and congenital disorders, particularly large segment defects exceeding critical dimensions, remain a major challenge in clinical orthopedics and regenerative medicine [1]. Statistics indicate millions of bone graft surgeries are performed globally each year, with demand continuing to rise amid accelerating population aging. Although autologous bone grafts possess osteoinductive, osteoconductive, and osteogenic properties, making them the gold standard for bone repair, their clinical application is limited by donor availability, the need for secondary surgery, and donor site complications. Allogeneic bone grafts avoid donor shortage issues, but carry risks of immune rejection and disease transmission. Furthermore, their osteoinductive activity is significantly reduced after sterilization processing. Synthetic metals and polymers, while scalable and mechanically tunable, generally lack biological activity and demonstrate suboptimal long-term efficacy [2,3]. Consequently, developing artificial bone graft materials with superior osteoinductive capacity remains an urgent challenge in bone-tissue engineering.

Mesoporous bioactive glass (MBG) is a novel biomaterial that has garnered significant attention in the field of bone repair in recent years. Compared to traditional bioactive glass, MBG possesses a highly ordered mesoporous structure, high specific surface area, and large pore volume. Research indicates that as a silicon-based material, MBG contains calcium–silicon components that release ions in simulated body fluid (SBF), inducing the formation of a hydroxyapatite-like layer on the material surface to promote calcium deposition [4,5]. Its ordered porous structure can also load specific biomolecules (such as growth factors and active proteins), enabling their sustained release at bone defect sites to effectively promote osteoblast differentiation and new bone formation [6]. However, the surface of unmodified MBG primarily consists of silanol groups, exhibiting relatively simple surface chemistry and lacking specific binding sites for osteoblast signaling molecules [7]. This nonspecific surface to some extent limits early cell adhesion and spreading on the material surface, while also weakening the material’s ability to precisely regulate cell behavior. To overcome this bottleneck, researchers have attempted surface functionalization modifications of MBG. R. M. et al. achieved sustained release of bone morphogenetic protein 2 (*Bmp2*) by loading it onto the MBG surface, thereby enhancing osteogenic activity and promoting new bone formation and repair at defect sites [8]. Y. Yang et al. designed an MBG/bovine serum albumin (BSA) composite bone graft material capable of continuously releasing bioactive ions, effectively promoting osteogenic differentiation and mineralization processes [9]. These studies demonstrate that MBG loaded with bioactive ions or functional proteins serves as an effective strategy to enhance bone repair outcomes. However, most current research focuses on classical growth factors like *Bmp2*, with limited investigations into the synergistic effects of other bone matrix proteins with MBG.

Osteopontin (OPN) is a multifunctional phosphoprotein widely distributed in the bone matrix. OPN contains an arginine, glycine and aspartic acid (RGD) sequence that is specifically recognized by integrin receptors on cell membranes, such as αvβ3, thereby mediating cell adhesion, spreading, and migration. Additionally, OPN contains an aspartic acid-rich region enabling its tight binding to hydroxyapatite in mineralized bone tissue to regulate calcium deposition. Studies indicate OPN also functions as a signaling molecule, activating multiple intracellular signaling pathways by binding to receptors such as integrins and Cluster of Differentiation 44 (CD44) [10,11]. Among these, the Erk1/2 signaling pathway has been confirmed as a core regulator of osteoblast differentiation. Studies demonstrate that OPN induces Erk1/2 phosphorylation, thereby activating downstream transcription factors (Runx2 and Osterix) and upregulating osteoblast-specific genes (e.g., *Bmp2*, *Collagen I*, and *Ocn*), ultimately promoting osteoblast differentiation and mineralization [12,13,14]. Therefore, we hypothesized that adsorbing OPN into the MBG-NH_2_ surface could synergistically enhance osteogenic differentiation and bone regeneration by both strengthening early osteoblast adhesion to the material and activating the Erk1/2 signaling pathway within the local microenvironment.

In this study, we first prepared MBG-NH_2_ and formed the MBG-NH_2_/OPN matrix through the physical adsorption of OPN. We evaluated the physicochemical properties of this material and investigated its effects on osteoblast adhesion, proliferation, differentiation and mineralization, and osteoblast-specific marker gene expression, while also exploring its molecular mechanisms. Our aim is to develop a new generation of bone repair materials for the treatment of bone defects and related diseases.

## 2. Materials and Methods

### 2.1. Substrates Preparation

MBG was fabricated through sol–gel and template self-assembly methods as described previously [15,16,17]. In detail, to prepare the MBG-NH_2_, Mg(NO_3_)_2_·6H_2_O and Ca(NO_3_)_2_·4H_2_O were used. The total molar amount of cations was controlled as 0.03 mol. First, 5.0 g poly(ethylene oxide-co-poly(propylene oxide)-co-poly(ethylene oxide)) triblock copolymer (P123) was dissolved in 300 mL sterile water and dropwise concentrated hydrochloric acid was used to adjust the pH to 1.0; then 7.0 g 1,3,5-trimethylbenzene (TMB) was added. The mixture was stirred at room temperature in an oil bath for 4 h. After that, 0.01 mol Mg(NO_3_)_2_·6H_2_O and 0.02 mol Ca(NO_3_)_2_ ·4H_2_O were added, with continued stirring in an oil bath (40 °C, 24 h). Furthermore, concentrated ammonia solution was dropwised to adjust the pH to 10.0, and the mixture was transferred to a reactor vessel, reacting in an oven (90 °C, 72 h). When the reaction was complete, the precipitate was collected after centrifuging at 1000 rpm for 15 min. The precipitate was washed with sterile water three times and dried at 100 °C. Next, the material was dried in a muffle furnace at 500 °C over 8 h, calcined for 6 h, and then cooled at a constant rate. Next, 0.1 g of the product was incinerated at 140 °C for 6 h. After natural cooling, 10 mL of 0.1 M 3-Aminopropyltriethoxysilane (APTES) was added and stirred in an oil bath at room temperature for 16 h for amino modification. Finally, the product was filtered and collected, sequentially washed and air-dried. An amino group was added to MBG to form MBG-NH_2._ (All the above reagents were procured from Macklin, Shanghai, China).

### 2.2. Characterization

The surfaces of MBG and MBG-NH_2_ were detected through a scanning electron microscope (SEM, JSM-6700F, JEOL, Tokyo, Japan). Mineralization deposits formed on the surfaces of MBG and MBG-NH_2_ after the materials were immersed in SBF medium (142.0 mM Na^+^, 5.0 mM K^+^, 1.5 mM Mg^2+^, 2.5 mM Ca^2+^, 103.0 mM Cl^−^, 27.0 mM HCO^3−^, 1.0 mM HPO_4_^2−^, and 0.5 mM SO_4_^2−^) for the indicated time [18]. Images of these mineralization deposits were recorded by a microscope (Leica, Wetzlar, Germany).

### 2.3. Cell Adhesion

The MC3T3-E1 cell line, a well established pre-osteoblast cell line originally derived from the calvaria of a newborn mouse, was kindly provided by Dr. Wang Zhao (School of Medicine, Tsinghua University, Beijing, China) and was cultured in Alpha minimum essential medium (αMEM, Gibco, Grand Island, NY, USA) supplemented with 10% fetal bovine serum (FBS), 100 units/mL of penicillin, and 100 μg/mL of streptomycin. MBG-NH_2_ was incubated with OPN (20 ng/mL) at 37 °C for 4 h to form MBG-NH_2_/OPN material. MC3T3-E1 cells were inoculated on these materials at a density of 2.5 × 10^4^ cells/well for at least 6 h. After 7 days of culture on these materials, MC3T3-E1 cells were washed, fixed with methylate-acetone (1:1) for 30 min, and blocked with 10% horse serum. Cell nuclei were stained with propidium iodide (PI, 0.1 mg/mL), and β-Actin was labeled using an anti-β-Actin primary antibody (Beyotime Biotech, Shanghai, China, 1:1000) conjugated with fluorescein isothiocyanate (FITC, 1:100, Beyotime Biotech, Shanghai, China). A laser microscope (Leica, Wetzlar, Germany) was used to obtain images.

### 2.4. Cell Proliferation, Differentiation and Mineralization

The proliferation of MC3T3-E1 cells was detected through Cell Counting Kit-8 (CCK-8, Beyotime Biotech, Shanghai, China) for 4 h at 37 °C before measuring under absorbance at 450 nm. The differentiation of MC3T3-E1 cells was determined according to alkaline phosphatase (ALP) activity through *p*-nitrophenol inorganic phosphate (PNPP) under absorbance at 405 nm. A bicinchoninic acid (BCA) protein assay kit (Pierce, Rockford, IL, USA) was used to detect protein concentration under absorbance at 550 nm. Relative ALP activity was calculated through the absorbance at 405 nm divided by the absorbance at 550 nm (A_405_/A_550_). The mineralization of MC3T3-E1 cells was determined by alizarin red solution (40 mM, pH 4.2) to stain calcium deposits. And these calcium deposits were identified by microscopes. The particle concentration employed in our experiments was 25 mg/mL.

### 2.5. Quantitative Real-Time Polymerase Chain Reaction (RT-qPCR)

Total RNA was obtained from MC3T3-E1 cells by Trizol reagent (Invitrogen, Carlsbad, CA, USA). After that, cDNA was synthesized with SuperScript III (Invitrogen, USA). The following forward and reverse primers were used to react quantitative real-time polymerase chain reaction (RT-qPCR) through a SYBR Green kit (Applied Biosystems, Carlsbad, CA, USA): *Glyceraldehyde 3-phosphate dehydrogenase* (*Gapdh*) 5′-CATGGCCTTCCGTGTTCCTA-3′ and 5′-CCTGCTTCACCACCTTCTTGAT-3′; *Bmp2* 5′-CTGACCACCTGAACTCCAC-3′ and 5′-CATCTAGGTACAACATGGAG-3′; and *Collagen I* 5′-CCTGGTAAAGATGGTGCC-3′ and 5′-CACCAGG TTCACCTTTCGCACC-3′ [19]. The program of PCR was as follows: first cycle (95 °C for 10 min, 56 °C for 1 min, 72 °C for 30 s), and next 44 cycles (95 °C for 30 s, 56 °C for 1 min, 72 °C for 30 s).

### 2.6. Statistical Analysis

Mean ± SE was used as a formula to express data that came from at least three independent experiments. Statistical significance was calculated by an unpaired Student’s *t* test. A threshold statistical significance was shown through a *p*-value of less than 0.05.

## 3. Results

### 3.1. Characterization of MBG and MBG-NH_2_

**Figure 1:**
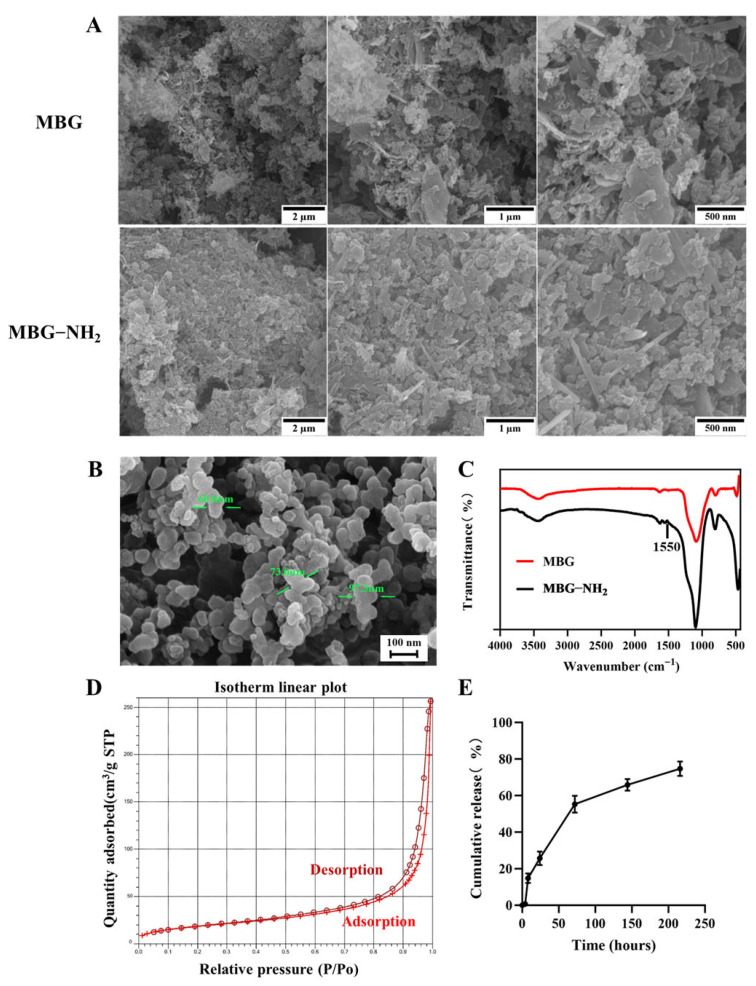
Characterization of materials. (**A**) SEM detection. (**B**) SEM detection of MBG-NH_2_ with a higher magnification, which showed the particle size of MBG-NH_2_. The green arrow marks the particle diameter. (**C**) Fourier transform infrared spectroscopy (FTIR) images of MBG and MBG-NH_2_ materials. (**D**) Brunauer–Emmett–Teller (BET) analysis of MBG and MBG-NH_2_ materials. (**E**) OPN release curve.

**Figure 2:**
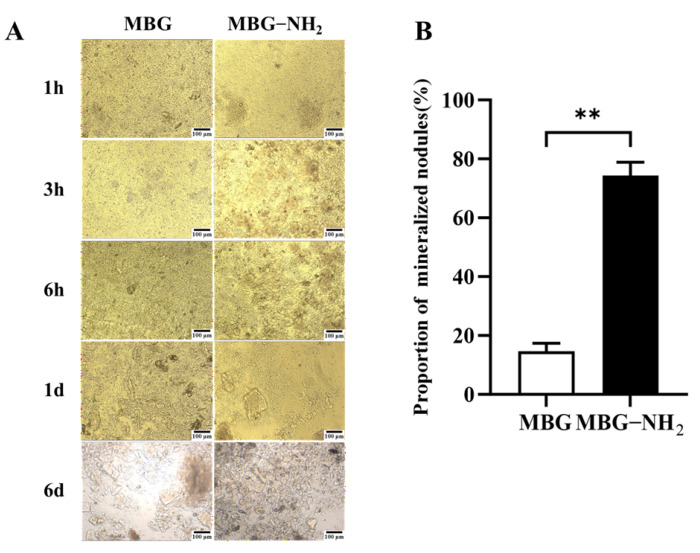
Mineralization capacity in vitro. (**A**) Morphology of mineralized deposits on MBG and MBG-NH_2_ materials at different time points in SBF buffer. (**B**) Quantification of Day 6 mineralized nodules. Statistical analysis was performed as indicated. ** *p* < 0.01.

To detect the surfaces of MBG and MBG-NH_2_ materials, we observed these surfaces through SEM. The results showed that they had exactly the same surface structures (Figure 1A). The particle size range of MBG-NH_2_ was approximately between 60 nm and 100 nm (Figure 1B). In addition, an absorption peak at 1550 cm^−1^ appeared in the FTIR spectrum of MBG-NH_2_ compared to MBG, which is characteristic of N-H bending vibrations, indicating that the -NH_2_ group had been successfully modified (Figure 1C). To verify the mesoporous structure of MBG, we conducted BET testing. The result showed a pronounced hysteresis loop, indicating that the synthesized material possessed a mesoporous structure (Figure 1D). According to these results, the surface area of MBG-NH_2_ was 68.02 m^2^/g, the pore diameter of MBG-NH_2_ was 22.15 nm, and the pore volume of MBG-NH_2_ was 0.40 cm^3^/g. We studied the release of OPN and found that the release rate of OPN was faster in the first 72 h, and the release amount of OPN reached 50%. The release rate became slower after 72 h, and the total release amount reached 75% at 216 h (Figure 1E). We also determined the calcium deposition rate of these materials immersed in SBF. We found that the bone-like deposition formed on the MBG-NH_2_ surface appeared earlier than it did on the MBG surface, and that more calcium was deposited on the MBG-NH_2_ surface than on the MBG surface (Figure 2A); quantitative analysis of mineralized nodules also support these results (Figure 2B). Taken together, these results indicated that MBG-NH_2_ was better than MBG in mineralization in vitro.

### 3.2. The Characterization of MC3T3-E1 Cells Cultured on These Materials

**Figure 3:**
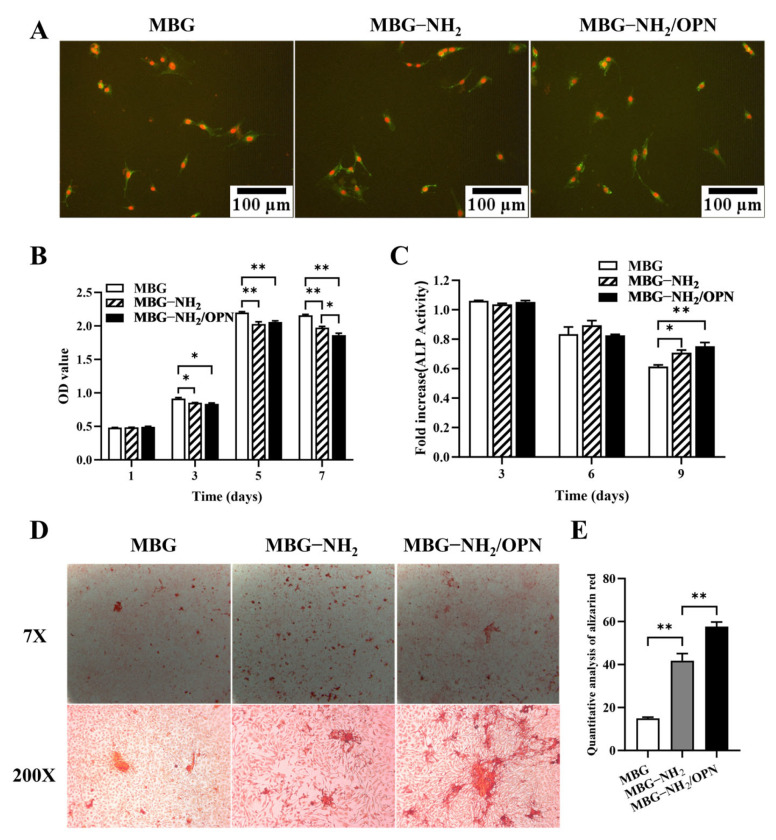
Osteogenic activity. (**A**) Cell adhesion of MC3T3-E1 cells on MBG, MBG-NH_2_, and MBG-NH_2_/OPN materials, respectively. PI stained nuclei (red) and FITC-labeled β-actin (green). (**B**) Proliferation of MC3T3-E1 cells cultured on MBG, MBG-NH_2_, and MBG-NH_2_/OPN materials for 1, 3, 5, and 7 days, respectively. (**C**) ALP activity of MC3T3-E1 cells cultured on MBG, MBG-NH_2_, and MBG-NH_2_/OPN material for 3, 6, and 9 days, respectively. (**D**) Mineralization of MC3T3-E1 cells cultured on MBG, MBG-NH_2_, and MBG-NH_2_/OPN material for 14 days, respectively. Mineralized structures observed under alizarin red staining (upper panel: 7× magnification; lower panel: 200× magnification). (**E**) Quantitative results of alizarin red staining. Statistical analysis was performed as indicated. * *p* < 0.05; ** *p* < 0.01.

The adhesion of MC3T3-E1 cells cultured on MBG and MBG-NH_2_ after seeding for 6 h are shown in Figure 3A. We found that more cells were adhered to the surface of MBG-NH_2_/OPN material, that there were more cells cultured on MBG-NH_2_ than on MBG material, and that MBG-NH_2_ material was better than MBG material for cell adhesion. However, the result of the CCK-8 assay showed that MC3T3-E1 cells cultured on MBG material proliferated faster than cells cultured on both MBG-NH_2_ and MBG-NH_2_/OPN materials (Figure 3B), which indicated that MBG material could enhance cell proliferation (none pattern column) and MBG-NH_2_/OPN material could suppress the proliferation of MC3T3-E1 cells (black column).

Interestingly, we also found that ALP activity was highly expressed in MC3T3-E1 cells cultured on MBG-NH_2_/OPN material compared with that in cells cultured on MBG-NH_2_ and MBG materials at Day 9 (Figure 3C). ALP is a marker in the early stage of osteoblast differentiation, and the expression of ALP decreases in the later stage of osteoblast differentiation, which was consistent with our results. In our results, ALP expression also decreased with time in MC3T3-E1 cells cultured on all these materials.

The third step of osteoblast in bone formation is matrix mineralization. In this study, to determine the matrix mineralization, we detected calcium deposition of the matrix in MC3T3-E1 cells cultured on these materials through alizarin red S. We found more calcium deposits in the matrix of MC3T3-E1 cells cultured on MBG-NH_2_/OPN material than in those cultured on MBG-NH_2_ and MBG material (Figure 3D). Quantitative results of alizarin red staining also confirmed this result (Figure 3E). Taken together, MBG-NH_2_/OPN material could enhance osteoblast adhesion, differentiation and mineralization, but it could suppress osteoblast proliferation.

### 3.3. The Expression of the Osteoblast-Specific Marker Gene

**Figure 4:**
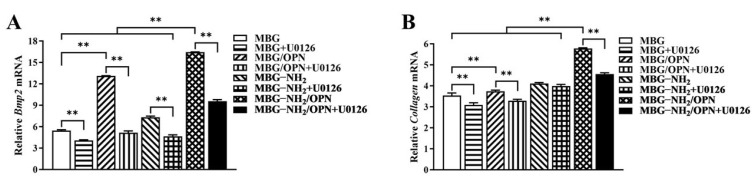
mRNA expression levels of osteoblast-specific marker genes in MC3T3-E1 cells cultured on MBG, MBG-NH_2_, and MBG-NH_2_/OPN materials. (**A**) *Bmp2.* (**B**) *Collagen I*. Statistical analysis was performed as indicated. ** *p* < 0.01.

To confirm the function of these materials to osteoblast, we detected the expression of the osteoblast-specific marker gene through real-time PCR. From our results, we found that the expression of *Bmp2* (Figure 4A) and *Collagen I* (Figure 4B) were higher in MC3T3-E1 cells cultured on MBG-NH_2_/OPN material compared with cells cultured on MBG-NH_2_ and MBG material. We also found that U0126 (an inhibitor of Erk1/2) could suppress the expressions of *Bmp2* and *Collagen I* in MC3T3-E1 cells cultured on all these materials. Thus, it is suggested that MBG-NH_2_/OPN material could enhance the expression of *Bmp2* and *Collagen I* in MC3T3-E1 cells compared to MBG and MBG-NH_2_ materials, and that MBG, MBG-NH_2_, and MBG-NH_2_/OPN materials activate the expression of *Bmp2* and *Collagen I*, possibly through the Erk1/2 activated pathway.

## 4. Discussion

This study evaluated the physicochemical properties of MBG-NH_2_/OPN material and its effects on osteogenic activity. We found that MBG-NH_2_ material formed earlier and more mineralized deposits than MBG material in SBF. We also observed that MBG-NH_2_/OPN material promotes cell adhesion, differentiation, and mineralization while simultaneously inhibiting cell proliferation. Notably, the osteogenic promotion of MBG-NH_2_/OPN material correlates with the upregulation of *Bmp2* and *Collagen I* gene expression, with the Erk1/2 signaling pathway also participating in this process. Collectively, these findings indicate that MBG-NH_2_/OPN has potential as a bone repair material.

SEM analysis revealed similar surface morphologies between MBG and MBG-NH_2_ materials, indicating that the amino modification did not disrupt MBG’s inherent mesoporous structure. Through BET analysis, we found that the pore size of the MBG-NH_2_/OPN material was approximately 22 nm. However, SEM observation revealed that these pore sizes were heterogeneous, and it was difficult to observe mesopores around 22 nm. This discrepancy primarily stems from the differing principles of the characterization techniques and specific morphological features of the sample; these two results are not contradictory. BET analysis calculates pore size distribution based on the adsorption behavior of nitrogen molecules within a material’s internal channels, providing a statistical average for the entire sample. However, mesopores around 22 nm, especially when they are closed or deep, are difficult to observe under SEM. Furthermore, SEM images only reflect the local two-dimensional morphology of a sample’s surface. Osteoid deposition occurred earlier and more extensively on the MBG-NH_2_ material surface. This may be attributed to the introduction of amino groups (-NH_2_) in MBG-NH_2_ material, which protonated to form positively charged (-NH_3_^+^) sites in SBF [20]. These sites more effectively attracted negatively charged phosphate ions (HPO_4_^2−^) in SBF, thereby accelerating calcium phosphate salt formation. At the cellular level, adding MBG-NH2/OPN particles to the culture medium resulted in rapid sedimentation and aggregation of most particles, forming a micro-nano-scale particle aggregate layer between cells and the culture dish, as shown in Figure 1B. This layer was not a dense structure, but possessed a certain porosity and roughness. This structure provided additional physical attachment sites for the extension, thereby enhancing cell adhesion efficiency. Furthermore, MBG-NH_2_/OPN particles altered the local microenvironment by releasing ions and proteins, indirectly promoting cell adhesion to the culture dish substrate. MBG-NH_2_/OPN material enhanced cell adhesion. This may have resulted from MBG’s amino group modification improving surface hydrophilicity and the charge environment, thereby facilitating the formation of a suitable adhesion matrix [16]. Additionally, physically adsorbed OPN can regulate cell adhesion behavior by binding to integrins on the cell surface, leading to increased numbers of adherent cells [14]. However, while it enhanced cell adhesion, MBG-NH_2_/OPN material relatively suppressed cell proliferation activity. This may have occurred because MBG-NH_2_/OPN material primarily promoted osteoblast differentiation, redirecting osteoblast energy from rapid proliferation toward osteogenic differentiation and mineralization. This also explains why, in the MBG-NH_2_/OPN material, despite slower absolute cell growth, both ALP activity and calcific nodule deposition were significantly higher than in other materials. In our culture system, MBG-NH_2_/OPN particles, being denser than the medium, rapidly settled to the surface of the culture dish under static conditions, forming a particle layer. Subsequently, BMSCs adhered to the culture dish with the particle layer. During fresh medium replacement, a portion of MBG-NH_2_/OPN particles were removed along with the medium, leading to a decrease in particle concentration. Therefore, we replaced the medium with fresh medium to minimize loss of the particle layer. Second, we ensured BMSCs underwent osteogenic differentiation by adding an excess of MBG-NH_2_/OPN particles during the initial cell seeding. Real-time quantitative PCR further corroborated these findings. Bmp2, a key bone-inducing growth factor, promotes osteoblast differentiation and maturation, participates in bone remodeling, and accelerates bone defect repair [21]; Collagen I, the primary organic component of the bone matrix, provides physical support for skeletal structures. The MBG-NH_2_/OPN material exhibited the highest mRNA expression levels for both genes, indicating successful activation of osteoblast-specific marker genes by the MBG-NH_2_/OPN material. Notably, we found that adding the Erk1/2 phosphorylation inhibitor U0126 to the medium suppressed the expression of *Bmp2* and *Collagen I*, suggesting that bioactive ions released by MBG (e.g., Si^4+^, Ca^2+^) and signaling molecules released by OPN may bind to specific cellular signaling sites, both activating intracellular Erk1/2 signaling pathways. In contrast, MBG-NH_2_/OPN material, by simultaneously releasing bioactive ions and OPN, maximally activated the Erk1/2 pathway compared to other materials. This likely drove the most efficient transcription of osteogenic genes, promoting osteoblast differentiation and mineralization. However, we also observed that both *Bmp2* and *Collagen I* expression levels were elevated in cells cultured on MBG-NH_2_/OPN substrates. Regarding *Collagen I*, a smaller decrease in expression levels was observed following treatment with the inhibitor U0126. We hypothesize that Collagen I, a key component of early osteoblast differentiation and the extracellular matrix, is regulated not only by the Erk1/2 signaling pathway, but also by the Transforming Growth Factor-beta/Mothers against decapentaplegic homolog (TGF-β/Smad) and Wingless-related integration site/Beta-catenin signaling (Wnt/β-catenin) pathways. When the Erk1/2 pathway is specifically inhibited by U0126, a compensatory mechanism is activated. OPN maintains basal type I collagen expression levels by activating the TGF-β/Smad and Wnt/β-catenin pathway, thereby ensuring extracellular matrix construction [22,23].

## 5. Conclusions

This study successfully developed an MBG-NH_2_/OPN composite material. This material exhibits significantly enhanced *in vitro* mineralization capacity and osteogenic induction activity. Notably, while promoting osteoblast adhesion, differentiation, and mineralization, it moderately suppresses excessive cell proliferation. Molecular mechanism studies indicate that this material activates the Erk1/2 signaling pathway and upregulates the mRNA expression level of *Bmp2* and *Collagen I* genes. Taken together, MBG-NH_2_/OPN effectively promotes osteoblast differentiation and mineralization. We propose that MBG-NH_2_/OPN has potential as a bone repair material for treating bone defect diseases.

## Data Availability

Original contributions presented in this study are included in the article. Further inquiries can be directed to the corresponding author.
